# Success Rates of Ankaferd Blood Stopper and Ferric Sulfate as Pulpotomy Agents in Primary Molars

**DOI:** 10.1155/2014/819605

**Published:** 2014-07-08

**Authors:** Kenan Cantekin, Hüsniye Gümüş

**Affiliations:** Department of Pediatric Dentistry, Faculty of Dentistry, Erciyes University, 38039 Kayseri, Turkey

## Abstract

*Purpose*. The purpose of this study was to evaluate clinical and radiographic findings of treatments using a new hemostatic agent (Ankaferd blood stopper (ABS)), as compared to ferric sulfate (FS), when used as a pulpotomy medicament in primary teeth. *Materials and Methods*. The primary molars (70) were selected from 35 children aged 4 to 6 years. The teeth were randomized into two groups for pulpotomy with the ABS (*n* = 35) and the FS (*n* = 35) agents. The patients were recalled for clinical and radiographic evaluation at 3-, 6-, 9-, and 12-month intervals. *Results*. At the 3- and 6-month clinical and radiographic evaluations, total success rates of 100% were observed in each group. In ABS and FS groups, the clinical success rates, however, reduced to 90.9% and 93.9% at the 9-month examination and 84,8% and 90.9% at the 12-month examination, respectively. Similarly, the teeth in the ABS and FS groups had radiographic success rates of 90.9% and 93.9% at 9 months and 84.8% and 87.8% at 12 moths, respectively. *Conclusion*. Although the findings indicated that ABS agents may be useful agents for pulpotomy medicament, further long-term and comprehensive histological investigations of ABS treatments are necessary.

## 1. Introduction

In primary dentition, pulpotomy is a common therapeutic procedure for the management of asymptomatic teeth with exposure caries [1]. Formocresol (FC), a devitalized agent, is the most widely used pulp medicament in the world. An FC solution at a 1 : 5 ratio is the gold standard in the pulpotomy of primary teeth because it is relatively simple to use, it is economical, and it has a high rate of clinical success [1, 2].

Although FC has a combination of favorable properties, the toxic, mutagenic, and carcinogenic effects of FC have led clinicians to use alternative methods and agents that are more biocompatible than FC [3–5].

Several factors affect the success of pulpotomy, and the hemostatic capability of the pulpotomy agent is one of the most important factors in improving the favorable prognosis of vital pulp therapy [6].

Ferric sulfate (FS) has been commonly used as a pulpotomy agent to control pulpal bleeding in vital pulp therapy for 30 years. FS induces hemostasis and the formation of a sealing membrane at the interrupted vessel of pulp tissue by agglutinating the blood proteins with ferric and sulfate ions [7].

Another potential type of hemostatic pulpotomy agent is the Ankaferd blood stopper (ABS; Ankaferd sağlık ürünleri AŞ, Istanbul, Turkey). The Ankaferd blood stopper is obtained from a herbal extract and is approved for use as a topical hemostatic medicine in dental surgery and external hemorrhage by the Turkish Ministry of Health. ABS provides a protein network by directly aggregating to erythrocytes without affecting the systemic circulation. Although ABS has been safely used to control bleeding in cases of epistaxis [8], after a tonsillectomy [9], to control gastrointestinal bleeding and bleeding by solitary rectal ulcer [10], there is very little information about the use of ABS as a pulpotomy agent in primary teeth. In the only clinical study conducted, Odabaş et al. [7] evaluated the effect of the application of ABS on the success of calcium hydroxide (CaOH) pulpotomies in primary teeth, and they reported that ABS + CaOH demonstrated a 95% total success rate at the 12-month followup.

The purpose of this randomized study is to compare clinical and radiographic findings of ABS, a new hemostatic agent, and FS as pulpotomy medicaments in vital primary teeth.

## 2. Materials and Methods

This randomized and split mouth study was conducted according to the Declaration of Helsinki. The study protocol was approved by the Ethics Board of the Medical Faculty of Erciyes University, Kayseri, Turkey.

Healthy, cooperative children were selected for the study from among the patients attending the clinic of the Department of Pediatric Dentistry, Erciyes University, Kayseri, Turkey. The primary molars (70) were selected from 35 children (18 boys and 17 girls) aged 4 to 6 years who had no medical condition that would contraindicate pulp therapy. Each child had at least two primary molars (first and/or second primary molar) with cariously exposed vital pulp requiring pulpotomy.

The participants were selected based on certain clinical and radiographic criteria.

The clinical criteria were as follows:

- a planned pulpotomy treatment for vital primary molars;
- tooth has involved at least 2 carious surfaces and can be restorable;
- no spontaneous or lingering provoked pain;
- the hemorrhage from the amputation site is bright red and easy to control.

The radiographic criteria were as follows:

- no evidence of internal or external root resorption;
- no evidence of intraradicular or periapical bone loss;
- there is no widening of the periodontal ligament space;
- no more than one-third of a physiological root resorption.

The primary molars were randomized into two groups for pulpotomy with the ABS (*n* = 35) or FS (*n* = 35) agents. Each pulpotomized molar was finished with a stainless steel crown.

A power calculation indicated that we needed 35 teeth in each group to demonstrate the effect at 92% power.

### 2.1. Treatment Techniques

The tooth was anesthetized using local anesthesia. Dental caries and overhanging enamel were removed with a #330 high-speed bur with a water spray. The same bur was used to gain access to the coronal pulp, and the entire roof of the pulp chamber was removed. A sharp discoid spoon excavator, large enough to extend across the entrance of the individual root canals, was used to amputate the coronal pulp. The pulp stumps were cleanly excised until the root canal orifices could be seen, with no tags remaining on the pulpal floor. Hemostasis was achieved at the amputation sites with water dampened cotton pellets, waiting for 5 minutes.

In the ABS and FS groups, ABS (Ankaferd sağlık ürünleri AŞ, Istanbul, Turkey) or FS (Viscostat, Ultradent Product Inc., Utah, USA) solutions applied on the pulp stumps with a dental syringe for 15 seconds, respectively, and the pulp stumps were rinsed with saline solution and pulp chamber was dried with sterile cotton pellets, subsequently. In both groups, a layer of reinforced zinc oxide eugenol paste (IRM, Densply, DeTrey, Konstanz, Germany) was placed into the pulp chambers and all the teeth were restored with glass ionomer cement (Equia, GC Europe, Tokyo, Japan). The final restoration with stainless steel crown (Unitek, 3M-ESPE, MN, USA) was made during the same appointment or within one week following the pulpotomy procedure. The patients were recalled for clinical and radiographic evaluation at approximately 3-, 6-, 9-, and 12-month intervals.

### 2.2. Clinical and Radiographic Examination

The criteria used for the clinical evaluation included a history of pain, tenderness to percussion, gingival abscess, sinus/fistula, and pathological mobility. The radiographic examination included an evaluation of internal/external root resorption, periapical/furcal radiolucency, and pulp canal obliteration. The teeth were considered to be radiographically successful in the absence of abnormal root resorption, internal root resorption, furcation involvement, and periapical bone destruction. Calcification in pulpal tissue and pulp canal obliteration were not regarded as defeat [11].

All pre- and postoperative clinical and digital radiographic examinations were performed at followup by one experienced investigator who was blind to the group being studied. The radiographic scores were compared for all the evaluated criteria using Pearson's chi-square analysis followed by Fisher's exact test (SPSS 17.0, SPSS Inc., Chicago, IL, USA) for the 3-, 6-, 9-, and 12-month followup periods. The significance level was set at *α* < 0.05.

## 3. Results

Sixty-four (91.4%) of the 70 restorations were evaluated at the 12-month followup examination; three patients did not return for the examination.

**Figure 1:**
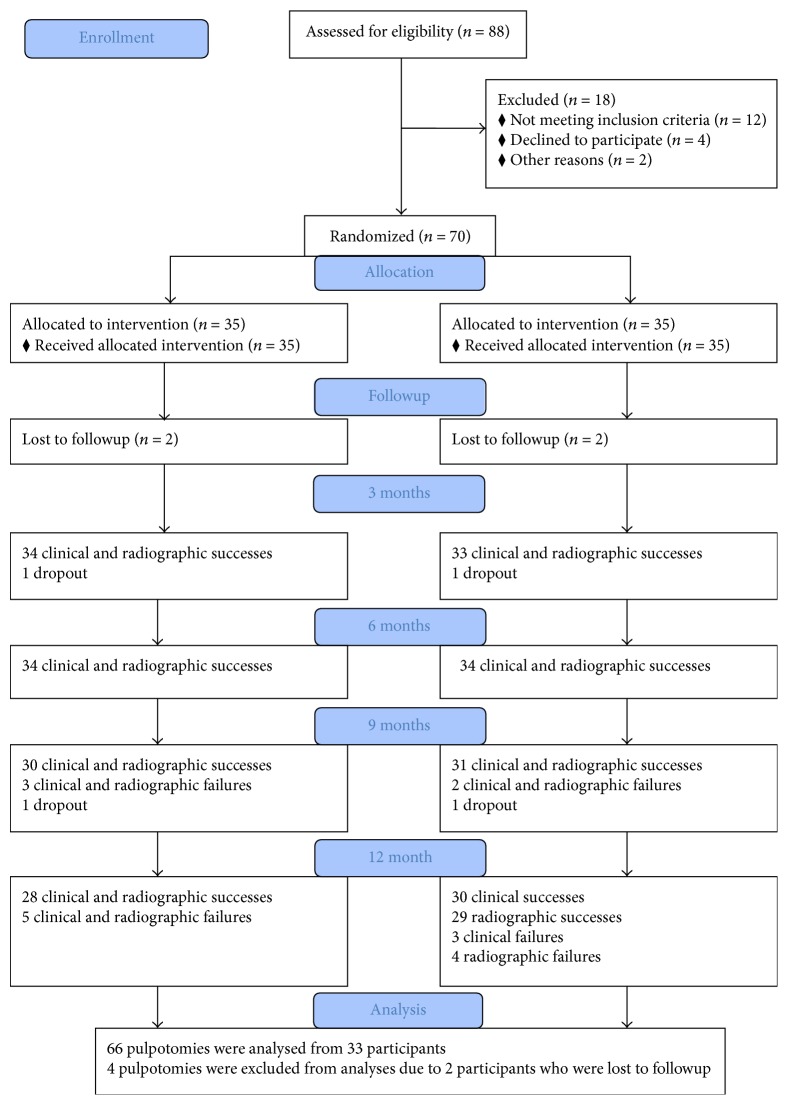
Flow of patients and pulpotomized teeth up to 12 months.

The clinical and radiographic findings of the experimental groups are presented in Table 1. At the 3- and 6-month clinical and radiographic evaluations, total success rates were observed to be 100% in each group (Figure 1).

ABS and FS groups had 100% clinical success rates, initially; however, the success rates reduced to 90.9% and 93.9% at the 9-month examination and 84.8% and 90.9% at the 12-month examination, respectively. Similarly, the teeth in the ABS and FS groups had radiographic success rates of 90.9% and 93.9% at 9 months and 84.8% and 87.8% at 12 months, respectively. Internal resorption and furcal radiolucency were the most common radiographic failure findings in both groups. No statistically significant difference (*P* > 0.05) between the ABS and FS groups was noted at each of the four followup periods.

## 4. Discussion

In this study, the clinical and radiographic success rates of pulpotomy using ABS treatments in comparison with FS treatments were examined over a 12-month period. Although the teeth treated with ABS demonstrated a slightly lower success rate (84.8%) than those treated with FS (87.8%), no statistically significant differences in success rates were found between the groups in the four examination periods (*P* > 0.05).

Formocresol is used by 92.4% of pediatric dentists and endodontists either in pure form or in a diluted solution at varying degrees of dilution [12]. Although FC pulpotomy is still the most commonly used pulp therapy for primary teeth, the carcinogenic potential of FC is a serious concern. The International Agency for Research on Cancer (IARC) has determined that formaldehyde causes nasopharyngeal cancer [13]. In addition, the use of FC as a pulpotomy medicament may lead to the development of an inflammatory response in the pulp, may be systemically distributed, and may lead to immunologic responses [14].

Ferric sulfate and mineral trioxide aggregate (MTA) have been proposed as an alternative pulpotomy medicament to FC in primary teeth in pediatric dentistry. MTA has some beneficial characteristics, such as being biocompatible and highly effective in preventing microleakage. MTA also promotes the regeneration of the original tissues when placed in contact with the dental pulp [14, 15]. Several* in vivo* and* in vitro* studies proved that MTA is a successful material when used as a pulpotomy agent in primary teeth. Most studies report that MTA has higher success rates than FC. Eidelman et al. [5] evaluated MTA and FC as pulpotomy agents for a 30-month period and reported that the MTA treatment produced a 100% success rate and that the FC treatment showed only one primary molar failure out of 32 molars. Aeinehchi et al. [16], Agamy et al. [11], and Erdem et al. [17] reported greater clinical and radiographic success rates for MTA compared with FC at 6-, 12-, and 24-month evaluations, respectively. Conversely, due to the high cost of MTA, it is unlikely that it will be used routinely for a pulpotomy in primary teeth in the near future, especially in a developing country. On the other hand, Schröder stated that formocresol as well as other nonbiological medicaments, including glutaraldehyde, Ledermix, and ferric sulfate, should be used in a restricted manner and confined to use in primary teeth [18].

In recent years, ABS has been proposed as an alternative pulpotomy medicament to FC and FS in primary teeth in pediatric dentistry. Several* in vitro* studies proved that ABS is an effective hemostatic and antibacterial agent in dentistry when used as a pulpotomy medicament in primary teeth. Çinar et al. [19] evaluated the antibacterial effect of ABS and FS on various oral microorganisms. They reported that FS and ABS exhibit not only hemostatic activity but also antimicrobial activity. Koyuturk et al. [20] evaluated the use of ABS as a pulpotomy agent in rat molars and compared it with FS and FC histologically. They reported that although there was no significant difference in inflammatory cell response between ABS and FS, a higher density of inflammatory cells was observed with FC. On the other hand, the FS mechanism involves the agglutination of blood proteins results from the reaction of blood with ferric and sulfate ions by forming plugs that occlude the capillary orifices [21, 22]. Therefore, FS has the potential to affect the overall coagulation factors for individuals; however, the ABS mechanism involves the formation of a protein network that acts as focal points for erythrocyte aggregation without affecting any individual factor [4]. In the only clinical study regarding the use of ABS as an pulpotomy agent, Odabaş et al. [7] evaluated the success of CH pulpotomy with and without ABS application. The CH group demonstrated a 90% success rate, and the CH + ABS group resulted in a 95% success rate; however, there was no significant difference between the two groups.

In several studies performed in human primary molars, FS demonstrated successful results when used as a pulpotomy agent. Papagiannoulis [23] and Ibricevic and Al-Jame [24] reported the success rates of 97% and 94% at 36 and 48 months, respectively. Smith et al. [25] reported radiographic and clinical success rates of 80% and 99%, respectively, at 19-month followup period. Erdem et al. [17] evaluated the success rates of MTA, FS, and FC as pulpotomy medicaments, and they reported total success rates of 96%, 88%, and 88% at the two-year followup, respectively. There was no significant difference among the groups. In the present study, the clinical and radiographic success rates of FS were approximately 90.9% and 87.8%, respectively, at the 12-month followup. The overall clinical success rates of FS in the aforementioned studies were found to be higher than the radiographic success rates. In the present study, the clinical and radiographic success rates were similar.

Hemostatic agents, particularly ferric sulfate, have been used as an alternative pulpotomy medicament to formocresol in primary teeth for 30 years. The Ankaferd blood stopper is a new hemostatic agent that contains natural herbal extracts and not only provides an ideal homeostasis on pulp tissue but also has an antibacterial effect. Additionally, the ABS differs from ferric sulfate in terms of the affect mechanism. Unlike FS, ABS does not have the potential to affect an individual's clotting factors. On the other hand, Bilgili et al. [4] assessed the hematological and biochemical safety in the oral administration of ABS in rabbits, and they reported that no signs of toxicity were observed after the administration. This is the first clinical study to compare the two hemostatic agents ABS and FS. The present study demonstrated that the ABS agent presented slightly lower clinical and radiographic success when compared with the FS agents at 12-month recall periods. The difference in success rates between hemostatic agents, however, was not statistically significant. This study demonstrates that the ABS agent is an attractive alternative to use as a pulpotomy medicament because it is biocompatible and yields successful results. Further long-term and comprehensive histologic studies of ABS treatments would be beneficial in determining the efficacy and success of this agent in pulpotomy treatment of primary teeth.

## 5. Conclusions

1. The Ankaferd blood stopper showed a similar clinical and radiographic success rate compared with ferric sulfate when used as a pulpotomy medicament for primary teeth during a 12-month evaluation period, and there was no significant difference in the groups.
2. Limited information exists in the research literature regarding the evaluation of ABS as a pulpotomy medicament* in vivo* or* in vitro*. Therefore, additional long-term clinical and histopathology studies are necessary.

## Figures and Tables

**Table 1:** Clinical and radiographic findings of two pulpotomy medicaments.

Findings	ABS (*N* = 33)				FS (*N* = 33)			
Clinical	**3**	**6**	**9**	**12**	**3**	**6**	**9**	**12**
No changes	33	33	30	28	33	33	31	30
Percussion	0	0	2	2	0	0	2	2
Swelling	0	0	0	1	0	0	0	1
Spontaneous pain	0	0	1	0	0	0	0	0
Fistula	0	0	0	2	0	0	0	1
Radiographic	**3**	**6**	**9**	**12**	**3**	**6**	**9**	**12**
Normal	33	33	30	28	33	33	31	29
Internal root resorption	0	0	0	3	0	0	1	3
External root resorption	0	0	0	0	0	0	0	0
Periapical radiolucency	0	0	0	0	0	0	0	0
Furcal radiolucency	0	0	2	2	0	0	1	2
Widened periodontal ligament	0	0	1	0	0	0	0	1
